# Adjusting the HIV prevalence for non‐respondents using mortality rates in an open cohort in northwest Tanzania

**DOI:** 10.1111/tmi.12304

**Published:** 2014-03-21

**Authors:** Filemon Tenu, Raphael Isingo, Basia Zaba, Mark Urassa, Jim Todd

**Affiliations:** ^1^ Amani Research CentreMuhezaTanzania; ^2^ Kilimanjaro Christian Medical University CollegeMoshiTanzania; ^3^ Mwanza Research CentreMwanzaTanzania; ^4^ London School of Hygiene and Tropical MedicineLondonUK

**Keywords:** HIV, prevalence, estimation, model, untested, mortality, VIH, prévalence, estimation, modèle, non testé, mortalité

## Abstract

**Objective:**

To estimate HIV prevalence in adults who have not tested for HIV using age‐specific mortality rates and to adjust the overall population HIV prevalence to include both tested and untested adults.

**Methods:**

An open cohort study was established since 1994 with demographic surveillance system (DSS) and five serological surveys conducted. Deaths from Kisesa DSS were used to estimate mortality rates and 95% confidence intervals by HIV status for 3‐ 5‐year periods (1995–1999, 2000–2004, and 2005–2009). Assuming that mortality rates in individuals who did not test for HIV are similar to those in tested individuals, and dependent on age, sex and HIV status and HIV, prevalence was estimated.

**Results:**

In 1995–1999, mortality rates (per 1000 person years) were 43.7 (95% CI 35.7–53.4) for HIV positive, 2.6 (95% CI 2.1–3.2) in HIV negative and 16.4 (95% CI 14.4–18.7) in untested. In 2000–2004, mortality rates were 43.3 (95% CI 36.2–51.9) in HIV positive, 3.3 (95% CI 2.8–4.0) in HIV negative and 11.9 (95% CI 10.5–13.6) in untested. In 2005–2009, mortality rates were 30.7 (95% CI 24.8–38.0) in HIV positive, 4.1 (95% CI 3.5–4.9) in HIV negative and 5.7 (95% CI 5.0–6.6) in untested residents. In the three survey periods (1995–1999, 2000–2004, 2005–2009), the adjusted period prevalences of HIV, including the untested, were 13.5%, 11.6% and 7.1%, compared with the observed prevalence in the tested of 6.0%, 6.8 and 8.0%. The estimated prevalence in the untested was 33.4%, 21.6% and 6.1% in the three survey periods.

**Conclusion:**

The simple model was able to estimate HIV prevalence where a DSS provided mortality data for untested residents.

## Abstract (fr)

**Objectif:**

Estimer la prévalence du VIH chez les adultes qui n'ont pas été testés pour le VIH en utilisant les taux de mortalité par âge et ajuster la prévalence globale du VIH dans la population incluant à la fois les adultes testés et non testés.

**Méthodes:**

Une étude de cohorte ouverte a été établie depuis 1994 avec le système de surveillance démographique (SSD) et cinq enquêtes sérologiques ont été menées. Les décès dans le SSD de Kisesa ont été utilisés pour estimer les taux de mortalité et les intervalles de confiance à 95% selon le statut VIH pour trois périodes de cinq ans (1995‐1999, 2000‐2004, 2005‐2009). En supposant que les taux de mortalité chez les personnes qui n'ont pas été testés pour le VIH soient semblables à ceux des personnes testées, et dépendent de l’âge, le sexe, le statut VIH et le VIH, la prévalence a été estimée.

**Résultats:**

En 1995‐1999, les taux de mortalité (pour 1000 personnes‐années) étaient de 43,7 (IC95%: 35,7 ‐ 53,4) chez les VIH positifs, 2,6 (IC95%: 2,1‐3,2) chez les séronégatifs et 16,4 (IC95%: 14,4 ‐ 18,7) chez les non testés. En 2000‐2004, les taux de mortalité étaient de 43,3 (IC95%: 36,2 ‐ 51,9) chez les séropositifs, 3,3 (IC95%: 2,8 ‐ 4,0) chez les séronégatifs et 11,9 (IC95%: 10,5 ‐ 13,6) chez les non testés. En 2005‐2009, les taux de mortalité étaient de 30,7 (IC95%: 24,8 ‐ 38,0) chez séropositifs, 4,1 (IC95%: 3,5‐4,9) chez les VIH négatifs et 5,7 (IC95%: 5,0 à 6,6) chez les non testés. Au cours des trois périodes d'enquête (1995‐1999, 2000‐2004, 2005‐2009), les prévalences ajustés de la période du VIH, y compris les non testés étaient de 13,5%; 11,6% et 7,1%, par rapport aux prévalences observées dans l'essai qui étaient de 6,0%; 6,8% et 8,0%. La prévalence estimée chez les non testés était de 33,4%, 21,6% et 6,1% dans les trois périodes de surveillance.

**Conclusion:**

Le modèle simple a été en mesure d'estimer la prévalence du VIH dans un cadre où un SSD a fourni des données de mortalité pour les résidents non testés.

## Abstract (es)

**Objetivo:**

Calcular la prevalencia de VIH en adultos que no se han hecho la prueba del VIH utilizando tasas de mortalidad específicas por edad, y ajustar la prevalencia total de VIH en la población para incluir tanto a adultos que se han realizado la prueba como a aquellos que no se la han hecho.

**Métodos:**

En 1994 se estableció un estudio de cohortes abierto con sistema de vigilancia demográfica (SVD) y desde entonces se han realizado cinco ensayos serológicos. Las muertes del SVD de Kisesa se utilizaron para calcular las tasas de mortalidad e intervalos de confianza del 95% según el estatus de VIH, durante tres periodos de cinco años (1995‐1999, 2000‐2004, y 2005‐2009). Se calculó la prevalencia de VIH asumiendo que las tasas de mortalidad en individuos sin prueba del VIH son similares a aquellos individuos que sí se han hecho la prueba, y dependientes de la edad, el sexo y el estatus de VIH.

**Resultados:**

Entre 1995‐1999, las tasas de mortalidad (por 1,000 personas años) fueron de 43.7 (IC 95% 35.7‐53.4) para VIH positivos, 2.6 (IC 95% 2.1‐3.2) en VIH negativos y 16.4 (IC 95% 14.4‐18.7) en sujetos sin prueba del VIH. Entre el 2000‐2004, las tasas de mortalidad eran 43.3 (IC 95% 36.2‐51.9) en VIH positivos, 3.3 (IC 95% 2.8‐4.0) en VIH negativos y 11.9 (IC 95% 10.5‐13.6) en sujetos sin prueba del VIH. Entre el 2005‐2009, las tasas de mortalidad eran del 30.7 (IC 95% 24.8‐38.0) en VIH positivos, 4.1 (IC 95% 3.5‐4.9) en VIH negativos y 5.7 (IC 95% 5.0‐6.6) en sujetos sin prueba del VIH. Durante los tres periodos del estudio (1995‐1999, 2000‐2004, 2005‐2009), la prevalencia ajustada de VIH para el periodo, incluyendo los sujetos sin prueba del VIH, eran 13.5%, 11.6% y 7.1%, comparado con la prevalencia observada en la población con una prueba de VIH del 6.0%, 6.8 y 8.0%. La prevalencia calculada en los sujetos sin prueba fue del 33.4%, 21.6% y 6.1% en los tres periodos de estudio respectivamente.

**Conclusión:**

Un modelo simple ha permitido calcular la prevalencia de VIH en donde los datos de mortalidad por SVD aportaron datos de mortalidad para residentes sin prueba de VIH.

## Introduction

The number of people living with HIV worldwide in 2007 was estimated at 33.2 million, with subSaharan Africa (SSA) being the most affected region (WHO 2008). Antenatal clinic (ANC) data obtained from pregnant women have been the main source of information for HIV prevalence trends and estimates of the number of people living with HIV (García‐Calleja *et al*. 2006). The use of ANC data has several constraints such as under representation of remote rural populations, lack of data on men and non‐pregnant women and limited ability to assess risk factors of HIV (Mishra *et al*. 2006).

In recent years, trends and population estimates of HIV prevalence in SSA are also estimated from household surveys, surveys among high‐risk groups (Islam & Conigrave 2008) and population‐based surveys such as the demographic and health surveys (DHS) and AIDS indicator surveys (Mishra *et al*. 2008). The advantage of using population‐based surveys is that they provide reliable and nationally representative direct estimates of HIV prevalence in countries with generalised epidemics as well as understanding of the magnitude and spread of epidemics (Mishra *et al*. 2008). Another advantage of population‐based surveys is that they can be a source of direct data on the distribution of HIV infection among different adult populations, men, women (pregnant & non‐pregnant) and across different geographical regions (Mishra *et al*. 2006).

The main challenge facing these surveys is a potential bias caused by non‐respondents which raises a major concern for analysis and generalisation (Nyirenda *et al*. 2010). The main reasons for non‐response in population‐based surveys are refusal to participate in HIV testing and absenteeism. Non‐response can bias population‐based estimates of HIV prevalence if it is systematically associated with HIV (Marston *et al*. 2008; Floyd *et al*. 2013). One method of overcoming the non‐response bias from population‐based surveys is using mortality rates to estimate HIV prevalence in non‐respondents (Nyirenda *et al*. 2010) and adjusting the overall HIV prevalence with a weighted average of the HIV prevalence in tested and untested residents.

This paper reports sex and age‐specific mortality rates in adults from a population cohort in north‐western Tanzania. The age‐specific mortality rates are used to adjust the population HIV prevalence for those who did not test for HIV.

## Materials and methods

### Data source

This analysis employed data collected since 1994 from a demographic surveillance study (DSS) in Mwanza Region in northwest Tanzania, as described by Mwita (Mwita *et al*. 2007).

### Study design

An open cohort study was established in 1994, and the population was enumerated every 6 months. Vital registration data on births and deaths were obtained, and verbal autopsy interview was used to interpret cause of death. Five serological surveys have been carried‐out in 1994–1995, 1997–1998, 2000–2001, 2003–2004 and 2006–2007. During these sero‐surveys, adults, aged 15–54 years, were asked to provide blood samples for anonymous HIV testing and responded to questionnaires covering sexual behaviour, use of health services and attitudes towards HIV (Urassa *et al*. 2001; Mwita *et al*. 2007).

### Definition of HIV status

HIV status was defined using data from all five HIV surveys between 1994 and 2007. Sero‐conversion date was obtained as the mid‐point between the last HIV negative test result and the first HIV positive test result. Residents were considered HIV negative from the date of their first HIV negative test until either the sero‐conversion date or 5 years after the last HIV negative test. Residents were considered HIV positive from the first HIV positive test or from the sero‐conversion date until 20 years after the last HIV positive test. Residents were considered of unknown HIV status prior to their first HIV test and for any period when they could not be defined as HIV positive or HIV negative according to the above definitions.

### Calculation of mortality rates

Total person years of follow up (PYO) was calculated from 1st January 1995 or the day that the individual entered the cohort through immigration or on their 15th birthday. All residents were followed up until the date of death, out‐migration or on their 55th birthday. For residents who out‐migrated and then returned to the DSS, the time when they were out of the DSS was not included in the total PYO. Mortality rate was calculated as the quotient of total deaths and PYO. Age‐specific mortality rates were calculated for three 5‐year intervals: 1995–1999, 2000–2004 and 2005–2009, these periods corresponding to a time of increasing HIV prevalence (1995–1999; Mwaluko *et al*. 2003), a time when HIV prevalence reached a plateau (Mwita *et al*. 2008), and a time of antiretroviral therapy (ART) availability (Wringe *et al*. 2012). Four age groups were used (15–24 years, 25–34 years, 35–44 years and 45–54 years), and for each period, mortality rates were calculated by age, sex and HIV status.

### Principal analytical model

For those with unknown HIV status, the HIV prevalence was estimated as the weighted average of the mortality rate of those with known HIV serostatus (Nyirenda *et al*. 2010). The calculation of the HIV prevalence was obtained for each group of sex (*s*), age group (*a*) and time period (*t*), using the formula

(1) $$H_{u}\left(s,a,t\right)=\frac{M_{u}\left(s,a,t\right)-M_{n}\left(s,a,t\right)}{M_{p}\left(s,a,t\right)-M_{n}\left(s,a,t\right)}$$

whereby, *M*_*u*_(*s,a,t*) = mortality in the unknown HIV, *M*_*n*_(*s,a,t*) = mortality in the known HIV −ve, *M*_*p*_(*s,a,t*) = mortality in the known HIV +ve, *H*_*u*_(*s,a,t*) = Prevalence in the unknown HIV.

For those with known HIV status, the HIV prevalence in each age, sex and time period *H*_*k*_(*s,a,t*) was estimated as the proportion of the person‐years lived by HIV positive persons, and was calculated as:

(2) $$H_{k}\left(s,a,t\right)=\frac{Y_{p}\left(s,a,t\right)}{Y_{p}\left(s,a,t\right)+Y_{n}\left(s,a,t\right)}$$

whereby, *Y*_*p*_(*s,a,t*) = Total person years lived by those tested positive, *Y*_*n*_(*s,a,t*) = Total person years lived by those tested negative.

For each age, sex and period, the adjusted HIV prevalence (*H*_*w*_(*s,a,t*)) was calculated as:

(3) $$H_{w}\left(s,a,t\right)=\frac{H_{u}\left(s,a,t\right)*Y_{u}\left(s,a,t\right)+H_{k}\left(s,a,t\right)*\left[Y_{p}\left(s,a,t\right)+Y_{n}\left(s,a,t\right)\right]}{Y_{p}\left(s,a,t\right)+Y_{n}\left(s,a,t\right)+Y_{u}\left(s,a,t\right)}$$

From this, the overall adjusted HIV period prevalence for each sex aged 15–54 years was obtained by multiplying the adjusted age prevalence of HIV by the proportion in each age band from a standardised population. To enable comparison between time periods, the standardised population was taken as the average from the Kisesa cohort across the whole time period 1995–2009.

### Ethical clearance

The research received ethical clearance from National Institute for Medical Research and approved by the Scientific and Ethical Review Committee of the National AIDS Control Programme of the Ministry of Health and Social Welfare, Tanzania. This work formed the dissertation project and was approved by the ethics review board at Kilimanjaro Christian Medical University College.

## Results

### Participation in HIV testing

Of eligible residents aged 15–54 years, the participation rate was 72.3% (6566/9078) in 1994/95 and 54.7% (7171/13 110) in 2006/7. Participation rates were lower in males (58%) than females (67.5%) for all serological rounds (Table 1).

**Table 1:** Participation rate in HIV testing among adults aged 15–44 years of age

Serology round	Males		Females		Both sexes	
	Tested	Percent	Tested	Percent	Tested	Percent
1994/1995	3092	70.2	3474	74.3	6566	72.3
1996/1997	3337	66.1	4011	74.0	7348	70.2
1999/2000	2991	55.4	3992	67.3	6983	61.6
2003/2004	3585	55.5	4536	64.2	8121	60.1
2006/2007	2992	47.7	4179	61.1	7171	54.7
Total	15 997	58.0	20 192	67.5	36 189	62.9

### Distribution of mortality rates by age, sex and HIV testing status

**Figure 1:**
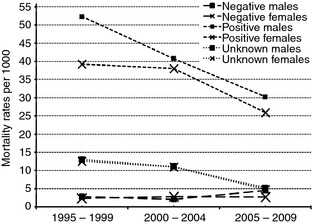
Trends of mortality rates in three survey periods.

Among residents aged 15–44 years of age, in the first 5 years of survey (1995–1999), 320 deaths were observed in 44 359 PYO, while in 2000–2004, 374 deaths were observed in 53 017 PYO, and in 2005–2009, 313 deaths in 59 502 PYO (Table 2). In HIV negative females, mortality rates across the three periods remained similar, while in HIV negative males, an increased mortality rate (4.6 deaths per 1000 PYO) was observed in the final period (2005–2009). HIV positive males and females had significantly higher mortality than HIV negatives, in all survey rounds (Figure 1). The overall mortality rate ratios (MRR) in females and males were between 13.6 and 19.4 for the first two periods (1995–1999 and 2000–2004). In the third period (2005–2009), the MRR had dropped to 6.6 in males and 9.3 in females (Table 2).

**Table 2:** Mortality rates and person years of follow up in HIV negative and HIV positive adults aged 15–44 years from 1995 through 2009

	HIV negative					HIV positive					MR ratio
	Deaths	PY	MR/1000	95% CI		Deaths	PY	MR/1000	95% CI		
				Lower	Upper				Lower	Upper	
1995–1999 – Males											
15–24	16	7681.8	2.1	1.3	3.4	1	143.3	7.0	1.0	49.5	3.3
25–34	14	4336.3	3.2	1.9	5.5	20	369.6	54.1	34.9	83.9	16.9
35–44	12	2850.5	4.2	2.4	7.4	20	269.6	74.2	47.9	110.0	17.7
Total	42	14 868.7	2.8	2.1	3.8	41	782.5	52.4	38.6	71.2	18.7
1995–1999 – Females											
15–24	13	6649.7	2.0	1.1	3.4	6	394.8	15.2	6.8	33.8	7.6
25–34	11	5445.1	2.0	1.1	3.6	31	596.6	52.0	36.5	73.9	26.0
35–44	13	3563.0	3.6	2.1	6.3	11	229.8	47.9	26.5	86.4	13.3
Total	37	15 657.7	2.4	1.7	3.3	48	1221.2	39.3	29.6	52.2	16.4
2000–2004 – Males											
15–24	8	7983.1	1.0	0.5	2.0	3	236.3	12.7	4.1	39.4	12.7
25–34	9	4442.9	2.0	1.1	3.9	16	365.9	43.7	26.8	71.4	21.9
35–44	16	3128.1	5.1	3.1	8.3	19	328.4	57.9	36.9	90.7	11.4
Total	33	15 554.1	2.1	1.5	3.0	38	930.6	40.8	29.7	56.1	19.4
2000–2004 – Females											
15–24	12	6965.3	1.7	1.0	3.0	8	409.7	19.5	9.8	39.0	11.5
25–34	14	5813.8	2.4	1.4	4.1	28	691.3	40.5	28.0	58.7	16.9
35–44	23	4427.6	5.2	3.5	7.8	20	367.8	54.4	35.1	84.3	10.5
Total	49	17 206.8	2.8	2.2	3.8	56	1468.8	38.1	29.3	49.5	13.6
2005–2009 – Males											
15–24	19	7119.7	2.7	1.7	4.2	1	212.0	4.7	0.7	33.5	1.7
25–34	14	3289.6	4.3	2.5	7.2	14	288.5	48.5	28.7	81.9	11.3
35–44	26	2461.1	10.6	7.2	15.5	10	325.2	30.7	16.5	57.1	2.9
Total	59	12 870.4	4.6	3.6	5.9	25	825.7	30.3	20.5	44.8	6.6
2005–2009 – Females											
15–24	6	5777.4	1.0	0.5	2.3	4	314.0	12.7	4.8	33.9	12.7
25–34	15	4806.4	3.1	1.9	5.2	14	636.7	22.0	13.0	37.1	7.1
35–44	19	3903.3	4.9	3.1	7.6	20	509.0	39.3	25.4	60.9	8.0
Total	40	14 487.1	2.8	2.0	3.8	38	1459.6	26.0	18.9	35.8	9.3

In the first two periods (1995–1999 and 2000–2004) for both males and females, the overall mortality for untested residents was 2–2.5 times as high as those who had tested for HIV (Table 3). In the last 5 years of survey 2005–2009, the mortality rates for tested and untested individuals were lower than previous years of survey. In the final period (2005–2009), the mortality rates in untested residents were similar to those in residents who had tested for HIV (Table 3).

**Table 3:** Mortality rates and person years of follow up in adults aged 15–44 years, who tested for HIV, and who did not test for HIV, from 1995 through 2009

	Did not test					Tested					MR ratio
	Deaths	PY	MR/1000	95% CI		Deaths	PY	MR/1000	95% CI		
				Lower	Upper				Lower	Upper	
1995–1999 – Males											
15–24	17	2650.0	6.4	4.0	10.3	17	7825.1	2.2	1.4	3.5	3.0
25–34	32	2117.2	15.1	10.7	21.4	34	4705.9	7.2	5.2	10.1	2.1
35–44	30	1287.6	23.3	16.3	33.3	32	3120.1	10.3	7.3	14.5	2.3
Total	79	6054.8	13.0	10.5	16.3	83	15 651.2	5.3	4.3	6.6	2.5
1995–1999 – Females											
15–24	24	3434.6	7.0	4.7	10.4	19	7044.5	2.7	1.7	4.2	2.6
25–34	31	1655.9	18.7	13.2	26.6	42	6041.7	7.0	5.1	9.4	2.7
35–44	18	683.9	26.3	16.6	41.8	24	3792.8	6.3	4.2	9.4	4.2
Total	73	5774.4	12.6	10.1	15.9	85	16 878.9	5.0	4.1	6.2	2.5
2000–2004 – Males											
15–24	24	3931.7	6.1	4.1	9.1	11	8219.3	1.3	0.7	2.4	4.6
25–34	35	3364.9	10.4	7.5	14.5	25	4808.8	5.2	3.5	7.7	2.0
35–44	46	2123.6	21.7	16.2	28.9	35	3456.5	10.1	7.3	14.1	2.1
Total	105	9420.2	11.1	9.2	13.5	71	16 484.7	4.3	3.4	5.4	2.6
2000–2004 – Females											
15–24	24	4883.2	4.9	3.3	7.3	20	7375.0	2.7	1.7	4.2	1.8
25–34	43	2557.0	16.8	12.5	22.7	42	6505.1	6.5	4.8	8.7	2.6
35–44	26	995.8	26.1	17.8	38.3	43	4795.4	9.0	6.7	12.1	2.9
Total	93	8436.0	11.0	9.0	13.5	105	18 675.6	5.6	4.6	6.8	2.0
2005–2009 *–* Males											
15–24	16	6244.6	2.6	1.6	4.2	20	7331.7	2.7	1.8	4.2	0.9
25–34	33	5594.8	5.9	4.2	8.3	28	3578.1	7.8	5.4	11.3	0.8
35–44	32	3489.0	9.2	6.5	13.0	36	2786.3	12.9	9.3	17.9	0.7
Total	81	15 328.5	5.3	4.3	6.6	84	13 696.2	6.1	5.0	7.6	0.9
2005–2009 – Females											
15–24	19	7687.7	2.5	1.6	3.9	10	6091.4	1.6	0.9	3.1	1.5
25–34	29	4666.3	6.2	4.3	8.9	29	5443.1	5.3	3.7	7.7	1.2
35–44	22	2176.6	10.1	6.7	15.4	39	4412.2	8.8	6.5	12.1	1.1
Total	70	14 530.5	4.8	3.8	6.1	78	15 946.7	4.9	3.9	6.1	1.0

### Adjusted HIV prevalence

Estimating the period prevalence of HIV from the mortality rates showed that HIV prevalence for adults who did not test for HIV in 1995–1999 was significantly higher across all age groups and sex. A total of 30 527 person‐years were contributed by adults who tested negative; and 2004 person years were contributed by HIV positive individuals, giving an observed period prevalence of 6.2% for tested residents in 1995–1999. The mortality for HIV negative individuals was 2.6 deaths per 1000 PYO, 44.4 deaths per 1000 PYO for HIV positive individuals and 12.8 deaths per 1000 PYO for individuals who did not test for HIV, giving a period prevalence of 24.5% for residents who did not test in 1995–1999. Taking into account the period prevalence of HIV and person years for HIV negative, HIV positive and untested individuals (11 829 PYO), the overall period prevalence of HIV adjusted for the whole population was 11.1% in the period of 1995–1999.

**Figure 2:**
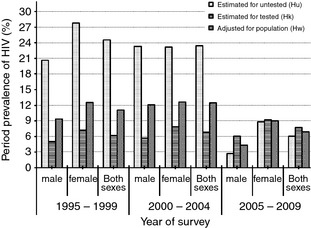
Period Prevalence of HIV by age and sex from 1995 to 2009.

In the period of 2000–2004, a total of 32 761 person years of follow up were recorded among HIV negatives, 2399 person years for HIV positives and 17 856 PYO for the untested, making an overall period prevalence of 6.8% for those who tested for HIV. The mortality rates (death per 1000 PYO) for HIV negatives, HIV positives and untested were 2.5, 39.2 and 11.1, respectively, giving a period prevalence of 23.4% for the untested. The adjusted period prevalence of HIV for the whole population was 12.4% in this survey period (Figure 2).

Individuals who tested negative for HIV in the time period of 2005–2009 were followed up for 27 357 PYO; HIV positives were followed up for 2285 PYO and untested for 29 859 PYO, making a period prevalence of 7.7% for the tested individuals. Using the overall mortality rates of 3.6 deaths per 1000 PYO for the HIV negative individuals, 27.6 deaths per 1000 PYO for HIV positive and 5.1 deaths per 1000 PYO for untested individuals, the estimated period prevalence of HIV for the untested residents was 6.0%. The weighted period prevalence of HIV for the tested and untested individuals was 6.9% in this time interval for adults aged 15–44 years (Figure 2).

## Discussion

Many studies conducted in subSaharan Africa (García‐Calleja *et al*. 2006; Marston *et al*. 2008; Nyirenda *et al*. 2010) have found poor participation in HIV testing particularly among males, giving rise to potential bias in the estimated HIV prevalence. We used the observed deaths and person years contributed by the residents in Kisesa DSS to adjust the HIV prevalence for the whole population in Kisesa. Based on the observed mortality, our analysis showed, in the period from 1995 to 2004, residents who did not test for HIV had significantly higher period prevalence of HIV than residents who tested for HIV across both sexes and all age groups. We calculated weighted period prevalence of HIV for the whole population and found that the weighted HIV prevalence was 80% higher than the observed HIV prevalence in both 1995–1999 and 2000–2005. However, for the period 2005–2009, we did not find increased mortality among those who had not tested for HIV, and the weighted HIV prevalence was 10% lower than that observed in those that tested for HIV in the surveys.

In this analysis, the proportion of non‐respondents increased in each subsequent serological survey, with a non‐participation rate of 28% in the first sero–survey and increased to 45% in the last sero‐survey. These findings are similar to rural South Africa (Nyirenda *et al*. 2010) and Malawi (Mishra *et al*. 2008). Several surveys mentioned reasons for not consenting for HIV testing as absenteeism (Marston *et al*. 2008; Reniers & Eaton 2009) and knowing that one is HIV positive already is less likely to participate, which may lead to underestimation of HIV prevalence (Floyd *et al*. 2013).

We investigated the pattern of mortality from 1995 to 2009 and found that mortality rates among HIV negative residents did not change much over the 15 years period. However, the mortality for HIV‐positive and untested decreased in each subsequent survey round. Although the mortality rate for HIV‐negative residents did not change significantly with time, mortality rates for HIV‐positive residents were 17 times higher than that of HIV‐negative residents in 1995–1999, 13 times higher than HIV negatives in 2000–2005 and eight times higher in 2005–2009 survey rounds. This was similar to the findings from South Africa, where the mortality rate for those who tested positive was 11–19 times higher than mortality in HIV‐negative individuals (Nyirenda *et al*. 2007). HIV‐positive males had slightly higher mortality rates than females in all survey years and mortality increased with age for both males and females. The proportion of Kisesa residents who die between the age of 15 years and 60 years was 49% and 46% for men and women, respectively, and attributed this increase of mortality to AIDS epidemic (Urassa *et al*. 2001). There has been a significant decrease of mortality for all residents in Kisesa cohort Michael *et al*. 2014). The decrease of mortality among HIV‐positive residents may be explained by the introduction of ART and better control and treatment of opportunistic infections.

Using DHS data from prior to the advent of ART in 14 African countries, including Tanzania, Mishra showed that untested males and females had significantly higher predicted prevalence of HIV than tested individuals (Mishra *et al*. 2008). Mishra did not use our model; instead, they used regression model to predict HIV prevalence among non‐testers using several indicators including, age, education, wealth index, residence, marital status, religion and geographic region. This study adapted the model that was previously applied to longitudinal data in KwaZulu‐Natal in South Africa by Nyirenda *et al*. (2010). We had similar findings to Nyirenda in the periods up to 2005, with HIV prevalence in all age groups higher in those who had not tested for HIV compared with those who had tested. However, in the period between 2005 and 2009, when ART became available to the population, there was little difference in the mortality rates and the estimated HIV prevalence between those who had tested for HIV and those who had not tested for HIV.

This analysis makes the assumption that among those who test and those who do not test, the mortality rates for HIV positive subjects are the same, and the mortality rates for HIV negative subjects are the same. In the final period of analysis (2005–2009), when ART became available in Tanzania, as expected, the mortality rates in those who did not test was higher than the mortality rates in the HIV negatives and lower than the mortality rates in the HIV positives. However, unlike in the previous periods, the estimated period prevalence of HIV in the untested was lower than the HIV prevalence in the tested. This could have been because some of those who did not take the anonymous test for HIV in our survey had previously tested HIV positive and because they knew their HIV status had been able to access ART, and thereby had improved survival over those who did test for HIV in our surveys. With the advent of ART, the mortality rates of HIV positive people who test and the mortality rates of HIV positive people who do not test differ, and the assumption behind this adjustment to the observed HIV prevalence does not hold.

In the first two periods, this model showed that if we do not take into account HIV prevalence in the un‐tested individuals, HIV prevalence estimated from individuals who consented to HIV testing can be underestimated. Similar findings have been documented from the analysis of eight DHS and one AIDS Indicator Survey in African countries (Marston *et al*. 2008). However, with the advent of ART, the relationship between HIV testing and mortality has changed, and the methods used in the analysis may not be useful to obtain an adjusted HIV prevalence in the population.

This model did not use detailed demographic data such as mobility, occupation, alcohol use, number of sexual partners, marital status, age at first sex, religion or any complex mathematical computations. It only used mortality and HIV testing status to estimate HIV prevalence for non‐respondents. The model has been commended by Nyirenda *et al*. in South Africa (Nyirenda *et al*. 2010) to be used in DSS settings across Africa, but this may not be suitable with the advent of ART. Other models used to estimate HIV prevalence for non‐testers such as multiple imputations (Barnighausen *et al*. 2008) and regression equations (Mishra *et al*. 2008) use detailed demographic characteristics which may suffer from reporting bias. In addition to that, detailed demographic characteristics are not uniformly collected in many DSS as compared to mortality data so relying on demographic data can lead to biased estimates (Nyirenda *et al*. 2010). One advantage of the mortality model is that it can be used whenever there is prospective mortality follow‐up data for individuals who participated in sero‐survey and those who did not participate even if there was only one sero‐survey conducted (Nyirenda *et al*. 2010). Moreover, researchers who opt to use this model do not need advanced mathematical expertise instead they just need to know simple arithmetic calculations to apply the model.

This model cannot be applied to the data from cross‐sectional studies where point prevalence is used as a measure of disease frequency. The model needs prospective adult mortality data for the calculation of the period prevalence of HIV in a specified population at risk that have the disease of interest over a specified period of time. Many national estimates of HIV prevalence uses data from cross‐sectional surveys which cannot be accommodated by this model (Islam & Conigrave 2008).

## Conclusion

This study has found poor participation in HIV testing among residents in Kisesa ward particularly among males, and the proportion of residents testing maintained gradual decrease for each subsequent year of sero‐survey. Mortality for non‐respondents was significantly higher than HIV‐negative residents. Moreover, mortality increased with age of residents and was slightly higher among untested males than untested females. HIV prevalence for non‐respondents was significantly higher in 1994–2004 survey years than prevalence of tested residents, and may provide an accurate adjustment for the calculation of the population HIV prevalence. However, with the advent of ART, this model may not allow an accurate adjustment for the population HIV prevalence.
